# Academic Impact of a Public Electronic Health Database: Bibliometric Analysis of Studies Using the General Practice Research Database

**DOI:** 10.1371/journal.pone.0021404

**Published:** 2011-06-22

**Authors:** Yu-Chun Chen, Jau-Ching Wu, Ingo Haschler, Azeem Majeed, Tzeng-Ji Chen, Thomas Wetter

**Affiliations:** 1 Department of Medical Informatics, Heidelberg University, Heidelberg, Germany; 2 School of Medicine, National Yang-Ming University, Taipei, Taiwan; 3 Institute of Pharmacology, National Yang-Ming University, Taipei, Taiwan; 4 Department of Neurosurgery, Taipei Veterans General Hospital, Taipei, Taiwan; 5 Department of Primary Care and Public Health, Imperial College London, London, United Kingdom; 6 Institute of Hospital and Health Care Administration, National Yang-Ming University, Taipei, Taiwan; 7 Department of Family Medicine, Taipei Veterans General Hospital, Taipei, Taiwan; Indiana University – Bloomington, United States of America

## Abstract

**Background:**

Studies that use electronic health databases as research material are getting popular but the influence of a single electronic health database had not been well investigated yet. The United Kingdom's General Practice Research Database (GPRD) is one of the few electronic health databases publicly available to academic researchers. This study analyzed studies that used GPRD to demonstrate the scientific production and academic impact by a single public health database.

**Methodology and Findings:**

A total of 749 studies published between 1995 and 2009 with ‘General Practice Research Database’ as their topics, defined as GPRD studies, were extracted from Web of Science. By the end of 2009, the GPRD had attracted 1251 authors from 22 countries and been used extensively in 749 studies published in 193 journals across 58 study fields. Each GPRD study was cited 2.7 times by successive studies. Moreover, the total number of GPRD studies increased rapidly, and it is expected to reach 1500 by 2015, twice the number accumulated till the end of 2009. Since 17 of the most prolific authors (1.4% of all authors) contributed nearly half (47.9%) of GPRD studies, success in conducting GPRD studies may accumulate. The GPRD was used mainly in, but not limited to, the three study fields of “Pharmacology and Pharmacy”, “General and Internal Medicine”, and “Public, Environmental and Occupational Health”. The UK and United States were the two most active regions of GPRD studies. One-third of GRPD studies were internationally co-authored.

**Conclusions:**

A public electronic health database such as the GPRD will promote scientific production in many ways. Data owners of electronic health databases at a national level should consider how to reduce access barriers and to make data more available for research.

## Introduction

Electronic health databases have been used widely in health research in recent decades. Such databases consist of data derived from routinely collected health records generated by daily clinical practice. With extremely large case numbers and long observation periods, electronic health databases have unique potentials in health research and can be very valuable if made available to academic researchers [1].

The United Kingdom's General Practice Research Database (GPRD) is one of the largest electronic health databases worldwide [2]. It was established in 1987 as the Value Added Medical Products (VAMP) Research Databank and transferred to the UK Department of Health in 1994, which has since then formally named the database as the General Practice Research Database (GPRD), ensured data quality, and made it available for academic research purposes [3].

Currently, the GPRD included around 5 million patients from around 590 primary care practices throughout the UK. Participating practices follow an agreed protocol for the collection of demographic and clinical data and submit anonymised records regularly using automated extraction to the database. The large number of patients included in the GPRD, and the accuracy and completeness of the data, make the database particularly suitable as a medical research tool [4]. In contrast to the health databases in other countires, which had restricted access and only limited contents, the UK made pioneering efforts to make GPRD publicily avialable to researchers not only in UK but also overseas. The potential postive influence on academic research could be anticipated [5]. However, the scope and scale of academic influence exerted by such electronic health databases have not yet been well investigated.

This study aimed to analyze GPRD related scientific productions in terms of the growth of publication numbers, patterns of co-authorship, study fields, and the academic impact of these GPRD publications. If the academic momentum achieved by a single public health database can be clearly demonstrated, the findings would serve as a reference for owners of large-scale electronic health databases in other countries and assist in future research.

## Results

### Growth of GRPD studies

The first GPRD study appeared as early as 1995, or one year after the GPRD was renamed and taken over by the Department of Health in England. A total of 749 GPRD studies published between 1995 and 2009 (assessed on April 19, 2010) were included in the analysis; earlier studies using the VAMP database were excluded from this analysis. By the end of 2009, the GPRD had attracted 1251 authors from 22 countries and been used extensively in 749 studies published in 193 journals across 58 study fields (Table 1). Most of the GPRD studies were articles (607, 81%) followed by meeting abstracts (15.5%), proceedings papers (2.9%) and letters (0.5%).

**Table 1:** Cumulated numbers and average annual growth rates of GPRD studies published between 1995 and 2009.

Year	Number of articles	Number of authors	Number of Subject Categories	Number of journals	Number of countries
1995	3	9	1	3	2
1996	7	16	4	6	2
1997	15	28	6	11	4
1998	35	64	10	19	7
1999	55	100	13	28	8
2000	98	179	28	49	11
2001	135	230	29	63	11
2002	181	303	31	77	13
2003	227	389	39	99	14
2004	304	508	44	123	15
2005	380	650	48	135	15
2006	464	805	53	150	18
2007	549	942	55	168	19
2008	648	1095	55	178	21
2009	749	1251	58	193	22
Average annual growth rate (%)	53.3	45.4	45.7	37.8	21.5

**Figure 1:**
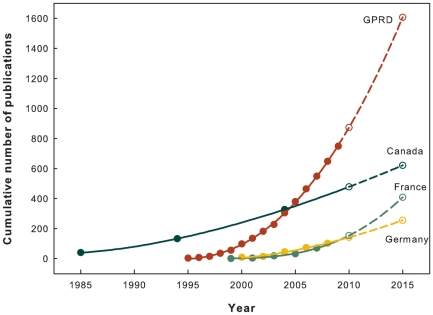
Cumulative numbers of GPRD studies compared with epidemiologic studies using public electronic health database in Canada, France and Germany. The cumulative numbers of studies published between 1995 and 2009 (solid data points) were fitted well with a power growth model (solid line). The predicted cumulative numbers of GPRD studies were then extrapolated by the fitted power model (hollow data points with short dashed line). The extrapolation should be interpreted cautiously under assumption. Data source: Germany: studies using German health insurance medication claims data [8]; France: studies using French reimbursement databases [9]; Canada: studies using Manitoba and Saskatchewan administrative health care utilization databases [10].

The number of GPRD studies increased substantially (Table 1). The average annual growth rate of GPRD studies was 53.3%, which was considerably higher than the 5.6% of all PubMed publications and 2.7% of all SCI records between 1997 and 2006 [6]. After the first GPRD study was published in 1995, the number of GRPD studies rapidly increased following a power law. Power growth model is an approved method to analyze the publication dynamics in scientific field [7]. The power growth model was fitted well with the numbers of the GPRD studies and showed an increased concave curve (R^2^ = 99.96%). Assuming all influential environmental factors remained the same for next 5 years, the number of GPRD studies would more than double in 2015 compared to 2009, with as many as 1646 expected publications. Fig. 1 shows past and extrapolated research dynamics of GPRD studies in comparison with studies using other public electronic health database derived from [8], [9], [10].

### Author productivity patterns

A total of 1251 authors had published 749 GPRD studies at the end of 2009. Almost all of the studies were co-authored (98.5%, 738/749). The number of authors per study ranged from one to 15, with a median of four. Since the publication of the first GPRD article in 1995, the most prolific author (Herschel Jick) had published 79 GPRD studies; the majority of authors (57.0%) had been involved in only one study until the end of 2009.

The advantages of conducting and publishing GPRD studies may increase further. The 17 most prolific authors (1.4% of all authors) contributed nearly half (47.9%) of GPRD studies; 65.6% of GPRD studies were produced by the 26 most prolific authors (3.6% of all authors) (Table 2). As the number of GPRD studies published increased, the number of authors producing these publications increased more slowly. Such skewed distribution suggests that the success of publishing GPRD studies was more reproducible in authors who had successfully previously published than those who had no or less experience in publication [11].

**Table 2:** Number of authors and GPRD studies with their cumulative percentage as grouped by number of authored GPRD studies published between 1995 and 2009.

Number of articles per author	Number of authors	Cumulative percentage to all authors (%)	Cumulative contribution to articles	Cumulative percentage to all GPRD studies (%)
20 or above	17	1.4	359	47.9
19-10	28	3.6	491	65.6
9-2	493	43.0	720	96.1
1	713	100.0	749	100.0

### Expansion in fields of study

The GPRD was applied in a growing number of study fields. In terms of the Subject Categories defined in SCI, the first GPRD study was published in field of “Medicine, General and Internal” [12]. Fifteen years later, the number of distinct fields of study had steadily increased to one-third of all SCI defined Subject Categories (33.4%, 58/172 Subject Categories) at the end of 2009.

The GPRD was most frequently used in study field of “Pharmacology and Pharmacy”, followed by “Medicine, General and Internal” and “Public, Environmental and Occupational Health” (26.4%, 14.2%, and 10.5%, respectively) (Table 3). The three most involved study fields accounted for half (51.5%) of all GPRD studies whereas the other half of GPRD studies were spread in 55 different study fields. These findings suggest that the GPRD had been used widely not only in the three aforementioned main streams but also in many other fields.

**Table 3:** Top 10 fields of study ranked by number of GPRD studies published between 1995 and 2009.

Field of study	Number of articles	Share of all GPRD studies (%)	Number of distinct journals
Pharmacology & Pharmacy	198	26.4	17
Medicine, General & Internal	106	14.2	20
Public, Environmental & Occupational Health	79	10.5	21
Gastroenterology & Hepatology	50	6.7	12
Endocrinology & Metabolism	47	6.3	11
Clinical Neurology	42	5.6	18
Rheumatology	39	5.2	7
Respiratory System	30	4.0	6
Obstetrics & Gynecology	25	3.3	9
Psychiatry	23	3.1	11

### Core journals and academic impacts

Because of the widespread study fields used in the application the GPRD, these papers had been broadly published in 193 journals by the end of 2009. The top ten journals published one-third (33.6%) of all GPRD studies (Table 4). The leading journal, “Pharmaco-epidemiology and Drug Safety”, had largest share, which was more than twice as many as the second journal, “The British Journal of Clinical Pharmacology”.

**Table 4:** Top 10 journals ranked by number of GPRD studies published between 1995 and 2009.

Journal name	Number of articles	Share of all GPRD studies (%)
PHARMACOEPIDEMIOLOGY AND DRUG SAFETY	99	13.2
BRITISH JOURNAL OF CLINICAL PHARMACOLOGY	39	5.2
BRITISH MEDICAL JOURNAL	23	3.1
PHARMACOTHERAPY	17	2.3
BRITISH JOURNAL OF GENERAL PRACTICE	15	2.0
EPIDEMIOLOGY	14	1.9
AMERICAN JOURNAL OF EPIDEMIOLOGY	12	1.6
GASTROENTEROLOGY	11	1.5
RHEUMATOLOGY	11	1.5
THORAX	11	1.5

GPRD studies were influential to successive studies in terms of citation counts. On average, GPRD studies were cited by more than 2 articles at the end of 2009 (average citations per article = 2.689). Most (84.4%) of GPRD studies were referenced by other studies (Table 5). Moreover, 45 GPRD studies have at least 45 citations; this showed both academic importance and productivity with the *h*-index of 45. Both finding suggested GPRD studies are influential in future research in their topic areas.

**Table 5:** Citation counts of GPRD studies published between 1995 and 2009.

Citation counts^a^	Number of articles	Share of all GPRD studies (%)
51–70	70	9.3
31–40	131	17.5
21–30	222	29.6
11–20	152	20.3
1–10	57	7.6
0	117	15.6

a Citation counts were calculated as numbers of articles that citing GPRD studies.

### Major national players

A paper is assigned to a country if it is listed as at least one author's affiliation. Accordingly, the UK is the most productive region of contributors: 52.6% of the papers had at least one UK author. Next is the USA with 41.7%. The ten most productive countries in terms of absolute number of papers are listed in the first column of Table 6. They account for 97% of the total number of GPRD studies (727/749 studies).

**Table 6:** Countries that published more than 15 GPRD studies between 1995 and 2009 ranked by the number of GPRD studies per million inhabitants.

Country	Number of GPRD studies	Number of GPRD studies per million inhabitants	Number of national SCI publications per 1000 inhabitants	Number of first authored-GPRD studies
UK	394 (1)	6.4 (1)	1.35 (5)	327 (1)
SWITZERLAND	45 (5)	5.9 (2)	2.80 (1)	38 (5)
NETHERLANDS	87 (3)	5.2 (3)	1.63 (3)	44 (4)
SWEDEN	43 (6)	4.7 (4)	1.97 (2)	5 (9)
SPAIN	70 (4)	1.7 (5)	1.00 (7)	54 (3)
CANADA	39 (7)	1.2 (6)	1.44 (4)	20 (6)
USA	281 (2)	0.9 (7)	0.99 (8)	209 (2)
FRANCE	21 (8)	0.3 (8)	0.93 (9)	9 (7)
ITALY	15 (10)	0.3 (8)	0.86 (10)	5 (9)
GERMANY	20 (9)	0.2 (10)	1.04 (6)	9 (7)

Values in parentheses are rankings. SCI, Scientific Citation Index.

To measure the research activity of GPRD studies, the absolute numbers of GPRD studies were adjusted to the population size of each country and the numbers of national SCI publications per thousand inhabitants were calculated as references (Table 6). The UK benefited most from GRPD studies, jumping from the 5^th^ most productive country according to SCI in general to the number one in GPRD studies. Switzerland and the Netherlands were also all active and productive in GPRD studies and took a clear lead over other countries. In consideration of the rank of first authored-GPRD studies (right most column in Table 5), Sweden appeared active but lacking of first authored papers.

### Internationally co-authored works

To study the degree of international collaboration, studies were divided into either internationally co-authored works or domestic works according to countries of authors' affiliation addresses. The national share of internationally co-authored works was calculated as the proportion of internationally co-authored works by all publications of a country. England, Scotland, Wales, and North Ireland were treated as one country. From 1995 to 2009, more than one-third (35.2%, 264/749) of GPRD studies were internationally co-authored. The rate of international co-authorship was high since before 2007, according to the international survey by [13], only 10 countries had reached such a degree of international work. The UK was involved in more than half (57.5%, 152/264 articles), which meant that nearly 60% of internationally co-authored GPRD studies had been co-worked with UK researchers.

**Figure 2:**
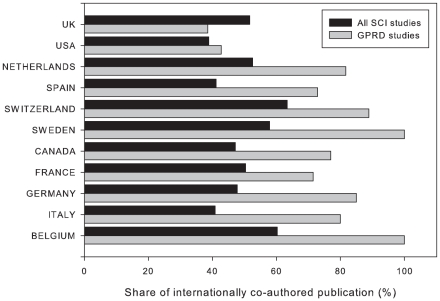
National share of internationally co-authored SCI studies and GPRD studies by countries. National share of internationally co-authored publications was calculated by the number of internationally co-authored work divided by the number of GPRD studies or SCI studies of the given country. As comparison reference values, the national share of internationally co-authored nationwide publications published in 2009 and indexed in the Scientific Citation Index Expand of given countries are plotted and abbreviated as SCI studies (Accessed 12 May 2010.).

Focusing on the 10 most active countries conducting GPRD studies (Table 6), the national share of internationally co-authored GPRD studies were substantially high. The national share of internationally co-authored GPRD studies of the listed countries, except the UK, was higher than that of their respective country baselines (Fig. 2). The international collaboration level of these listed countries was between 40% and 60%, much higher than that of other countries, thus making the proportion of internationally co-authored GRPD studies higher. Sweden and Belgium had the highest share (100%) of internationally co-authored GPRD studies. This indicated that all of Sweden and Belgium's GPRD studies between 1995 and 2009 were internationally co-authored. Germany (93%) followed by Switzerland (83%), Spain (73%), France (65%), Canada (64%), and Italy (63%) were also active in conducting GPRD studies internationally.

The largest contributors of GPRD studies, the UK, had the lowest national share of international collaboration. This was because of the size effect of its domestic GPRD studies. Despite the large number of internationally co-authored studies, there were still many GPRD studies produced by UK researchers alone.

## Discussion

The GPRD is used widely internationally. However, its influence had not been well investigated. This study extracted GPRD studies published between 1995 and 2009 from the SCI and analyzed the growing influence of GPRD studies from four different perspectives. In terms of number of studies, the rapid increase is in a power growth fashion. Observing author productivity patterns, the findings suggest that the success of conducting GPRD studies is self-sustaining. The GPRD is used widely in numerous study fields and not limited to general medicine. It is also used actively by international co-workers. Most importantly, GPRD studies showed substantial influence on successive studies.

There are some limitations in this study. First, the analysis aimed at demonstrating the resulting impact of GPRD as a publicly available research material. The observation is primarily based on historical data during last 15 years. Since there are many factors that may substantially influence scientific production, interpretation or extrapolation of our data should be done with caution. Second, to make the results comparable, only GPRD studies indexed in the Thomson's SCI were included in the analysis. This considerably underestimated the overall scientific output related to GPRD. If un-indexed studies, such as posters and abstracts, were included, the output related to GPRD should be much higher. Third, only studies that mentioned “GPRD” were included, but not those that used its antecedent, the VAMP Research Databank. If the latter was taken into account, the earliest publication could be traced as early as 1988, according to the GPRD bibliography [14].

The UK's GPRD, with its extremely large patient numbers and long observation period, is becoming important in health research [15], [16]. The increase in number of GPRD studies is dramatic, compared to a previous study [17] and the number of scientific outputs usually doubles every 10 to 20 years. The authors are not aware of any scientific discipline with this order of magnitude of duplication time of only six years. Similarly, rapid growth is also reported in epidemiologic studies using electronic health databases worldwide, such as Medicare data in the US [18], Manitoba and Saskatchewan province database in Canada [10], National Health Insurance Research Database (NHIRD) in Taiwan [19], [26], and health insurance data in Germany [8], and France [9]. The findings suggest that electronic medical record databases are used actively in current health research.

This study clearly demonstrates academic benefits from the GPRD. While most electronic health databases are used in pharmaco-epidemiologic studies [20], the GPRD has not limited its use to a single field. As an electronic medical record system, it includes basic demographic details, medical records, drug history, and prevention records. In addition to its comprehensive contents, linkages to external registries are also made possible by sophisticated encryption procedures [4]. Furthermore, substantial efforts have been made to improve data quality and validity [21]. These characteristics make the results of GPRD studies comparable to clinical trials [22] and explain GPRD's widespread utilization in various study fields.

Considerable efforts are needed to use GPRD for academic research. Since the GPRD is primarily designed for data captured from daily clinical practice, researchers should be highly knowledgeable about each step of the data collecting process and carefully tailor the data for their research problems [21]. This provides an explanation why most GPRD studies are results of co-work and the unbalanced distribution of authors' patterns of outputs. Researchers are prone to work closely with knowledgeable people about every detail of GPRD to share technology and experience [20], [23]. Attempts are now being made to overcome technical barriers to conduct more GPRD studies [24], [25]. If these efforts make GPRD more accessible to researchers, an even more prominent growth in GPRD studies can be expected.

Our analysis demonstrated that a small percentage of authors contributed to the majority of GPRD studies. A closer look of these highly productive groups and authors shows that the value of GPRD's public availability has boosted their productivity. GPRD studies are an alternative approach to achieve as meaningful results as clinical trials without the necessity to invest the good clinical practice (GCP) driven efforts necessary for each and every new experimental trial [20]. Moreover, GPRD studies and studies of similar origin improve in efficiency and efficacy with every successful study established. As automatic procedures are conceived and shared among cooperating institutions, each individual study competence builds. Therefore the costs and manpower of each study will likely to decrease. In other words, the “success breeds success” phenomenon which in its original interpretation in scientometrics emphasizes individual competence correlates to related individual success [11] transforms into a societal “success breeds success” phenomenon: society gets out ever more once a scientific community has got “over the hump” of mastering the initial intricacies of getting the right data in the right quality out of a health care delivery (such as GPRD) or claims database [26].

The most distinguishing feature of GPRD from other nationwide electronic health databases is its international co-author pattern. The analysis shows that the GPRD is used actively by means of international co-work. Such a high share of international co-authorship reflects a universal demand for large scale electronic health databases. This should encourage national- or institutional-level data holders to consider re-using their electronic health databases for academic purposes. Moreover, an international strategy not only effectively promotes academic activities but also provides important opportunities for the reciprocal exchange of research knowledge among countries [4].

### Conclusion

Based on a quantified analysis of the influence of GPRD use, a public electronic health database can promote scientific production in many ways. To promote academic research and health care, data owners of electronic health databases at a national level should consider how to reduce access barriers and to make data more available for research. The increasing use of electronic patient records worldwide will make more data available for secondary analysis, subject to legal and technical challenges in accessing clinical data being addressed. These data have great potential as research tools and can help in maximizing the outputs from the large investments being made by many health systems in the development of electronic patient records and eHealth systems.

## Materials and Methods

To measure the scientific production exerted by the GPRD, studies used GPRD were extracted and analyzed. Publications from 1990 to 2010 in the Science Citation Index (SCI) of the Thomson Scientific's Institute for Scientific Information (also known as Thomson's ISI, Philadelphia, PA, USA) were used as the main data source. Documents recorded as article, letter, note, proceedings paper, or meeting abstract were considered. Publications with GPRD or “General Practice Research Database” as their topics were defined as GPRD studies and extracted for further analysis. Publications that used the VAMP Research Databank, the precursor of GPRD, were not included in the analysis.

Each GPRD study was assigned into at least one study field based on the article's Subject Categories defined in the SCI. Authors were identified using the author names in last-name-plus-initial form as recorded in the SCI. Papers were assigned to countries according to the affiliation addresses of authors. All countries listed in the address were considered. A paper was considered as an internationally co-authored work if assigned to more than one country. National share of internationally co-authored publications was calculated by the number of internationally co-authored work divided by the number of GPRD studies of the given country.

To measure the academic impact of GPRD studies to successive studies, citation counts at the end of 2009 were included in this analysis. Average citation count per publication was calculated by the number of publications that citing these GPRD studies divided by the number of GPRD studies. The H-index is also calculated to demonstrate the importance and productivity of GPRD studies [27].

To compare with the national shares of publications across countries in relation to country sizes, the number of SCI publications and internationally co-authored SCI publications of given countries in 2009 were obtained from the Web of Science®. The number of inhabitants per country in 2009 was extracted from the World Factbook 2009, the official statistic report from the US government [28]. Microsoft SQL server 2008, together with Microsoft Excel 2007, was used for data processing and calculations.
